# Optimization of Self-Directed Target Coverage in Wireless Multimedia Sensor Network 

**DOI:** 10.1155/2014/416218

**Published:** 2014-06-30

**Authors:** Yang Yang, Yufei Wang, Dechang Pi, Ruchuan Wang

**Affiliations:** ^1^College of Computer Science and Technology, Nanjing University of Aeronautics and Astronautics, Nanjing, Jiangsu 210016, China; ^2^Department of Information Technology, Nanjing Radio and TV University, Nanjing City Vocational College, Nanjing, Jiangsu 210002, China; ^3^College of Computer, Nanjing University of Posts and Telecommunications, Nanjing, Jiangsu 210003, China

## Abstract

Video and image sensors in wireless multimedia sensor networks (WMSNs) have directed view and limited sensing angle. So the methods to solve target coverage problem for traditional sensor networks, which use circle sensing model, are not suitable for WMSNs. Based on the FoV (field of view) sensing model and FoV disk model proposed, how expected multimedia sensor covers the target is defined by the deflection angle between target and the sensor's current orientation and the distance between target and the sensor. Then target coverage optimization algorithms based on expected coverage value are presented for single-sensor single-target, multisensor single-target, and single-sensor multitargets problems distinguishingly. Selecting the orientation that sensor rotated to cover every target falling in the FoV disk of that sensor for candidate orientations and using genetic algorithm to multisensor multitargets problem, which has NP-complete complexity, then result in the approximated minimum subset of sensors which covers all the targets in networks. Simulation results show the algorithm's performance and the effect of number of targets on the resulting subset.

## 1. Introduction

Wireless multimedia sensor network (WMSN) [1], an advancing form of wireless sensor network (WSN) [2], is a multihop self-organizing distributed sensing network which is constituted by battery of multimedia sensor nodes with ability of calculation, storage, and wireless communication. Through multimedia sensor in nodes, this advanced network can find, collect, and handle many kinds of media information, such as audio, video, and image, in surrounding environment. Then the multimedia data will be sent to information gathering center by multihop trunking scheme. After data analysis in information gathering center, overall and effective environment monitoring will be accomplished [1, 3].

One of the most important issues in WSN is sensing coverage, which is also measurement for service quality in WSN [4]. In traditional sensor network, the sensor coverage is usually hypothesized as omnidirectional and predefined as a crude round [1]. But, in WMSN, the coverage method is very different with its traditional sensor network. The nodes in WMSN, such as video and image sensor nodes, catch a directed visual field, named as field of view (FoV) [5]. Usually the hypothesis is that direction of multimedia sensor node is adjustable [6]. When the node is randomly deployed into malicious environment, it can cover the target and the area self-directly through cooperation with neighbor nodes.

In this paper, the coverage expectation value from node to target is delineated through probabilistic models of FoV disk, which is the ground for self-direction wireless multimedia sensor node and better coverage of target. The optimization algorithms for single-node single-target, multinode single-target, and single-node multitarget are given to solve the coverage problem. Also, the self-directed direction of target covered by FoV disk for every node is set as candidate sensor direction. Genetic algorithm is used to discriminate the self-directed coverage optimization in multinode multitarget circumstance and to discover the minimum set of nodes for coverage of all targets.

## 2. Relevant Work

Coverage issue of randomly deployed sensor node is the hotspot in this field. In traditional WSN, most researches hypothesise that the omnidirection sensor node which covers area is a circle with its center in node. But, in WMSN, the sensor cover area is usually hypothesized as sector, which is not applicable for traditional coverage algorithm [6, 7]. Coverage issue can be divided into two categories, the area coverage which ensures that the whole area is covered by sensor node and the target coverage which ensures that every interested target in district is covered by at least one sensor node.

New distributed algorithm has been raised after research in self-directed WMSN [6]. This algorithm realizes node optimal sensing direction, minimal node coverage redundancy in sensing field, and maximal multimedia coverage. Directional sensor network deployment with connectivity and coverage guarantee has been considered in recent study [8]. In this study, minimal directional sensor network was deployed to form a connected network covering interested area. For the target coverage in directional sensor network, different priority was assigned to target [7]. Taking into account the distance between the target and node, a problem of target coverage based on priority has been suggested and a minimal node set was tried to be chosen for all targets monitoring.

In target coverage problem of directional sensor network, the direction and rotation of angle in node directional compensation were not considered [9]. Also, for resolving multinode multitarget coverage optimization, a battery of fixed sensing direction was set up for every sensor node, which was related to node angle of sensing [9]. But rotatable self-directed sensor node has 360° sensing direction. In this research, the self-directed direction and angle of rotation are inducted into self-direction process of multimedia sensor network node, which could decrease the adjustment of sensor node to save energy while optimizing target coverage.

## 3. Network Hypothesis and Multimedia Coverage FoV Model

The network field in our research is two-dimensional Euclidean field with randomly distributed multimedia sensor nodes and a certain number of interested targets, which means that the sensing direction and position of all nodes are random and independent. The same as the hypothesis in recent study [6], the image senor is equipped with multimedia sensor node to provide FoV with angle value Θ, which could bilaterally rotate to redefine sensing direction in two-dimensional Euclidean area. Simultaneously, it is hypothesized that monitoring target and multimedia sensor node can achieve their position information through lightweight positioning technology of WSN [10]. Also the nodes in network are hypothesized to have same sensing model, which means that they have identical sensing radius and angle. Since the WMSN is self-oriented, the sensor nodes constituting network can regulate their sensing angle, which means that inexpensive multimedia node can rotate around its vertical axis [6].

In a WMSN, *S* = {*s*_1_, *s*_2_,…, *s*_*N*_} is the set of multimedia sensor nodes, *R*_*s*_ is sensing radius of node, and interested target battery is *T* = {*t*_1_, *t*_2_,…, *t*_*m*_}. With regard to sensing model of multimedia sensor node, the definition is provided as follows.

**Definition 1 .**

**Figure 1:**
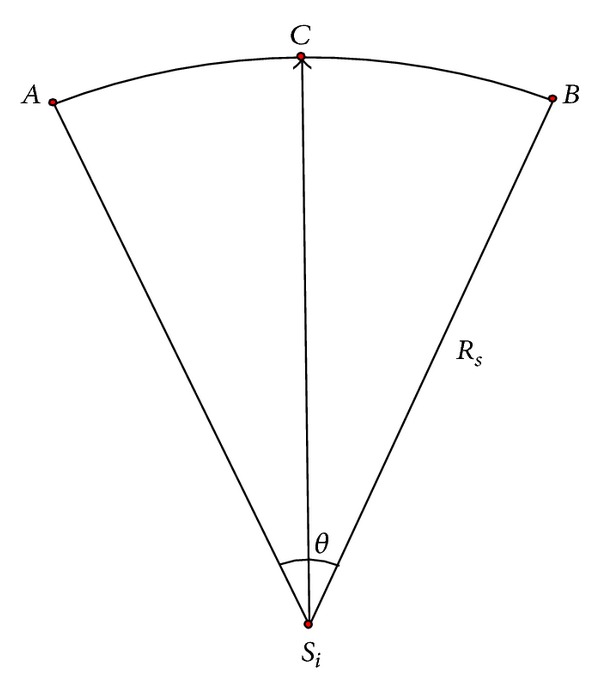
2D FoV sensing model.

2D FoV: 2D FoV is a directional sensing area of multimedia sensor node, which is hypothesized as a proximate sector in two-dimensional space (Figure 1). 2D FoV of node *s*_*i*_ is defined as tetrad (*s*_*i*_, *R*_*s*_, *θ*, *s*_*i*_ *C*), with *s*_*i*_ as sensor, *R*_*s*_ as sensing radius, *θ* as vertex angle of sector, and *s*_*i*_ *C* as current sensing direction of node *s*_*i*_ which divide sector into two halves.

**Definition 2 .**

**Figure 2:**
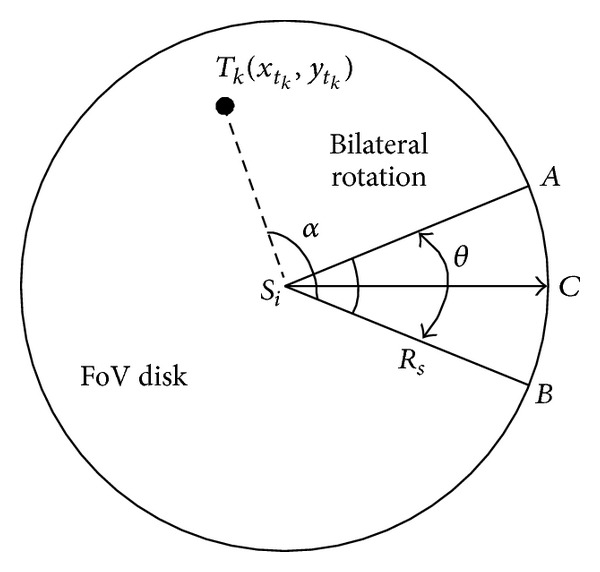
FoV disk model.

2D FoV disk: the 2D FoV disk of multimedia sensor node is defined as a set of all possible 2D FoV of node, which should be a round area with radius as *R*_*s*_ (Figure 2).

**Definition 3 .**

Target coverage of multimedia sensor node *C*_*T*_*k*__(*s*_*i*_): equation (1) shows the coverage *C*_*T*_*k*__(*s*_*i*_) of node *s*_*i*_ to target *T*_*k*_:

(1) $$\begin{array}{c} C_{T_{k}}\left(s_{i}\right)=\{\begin{array}{cc} 1, & d\left(s_{i},T_{k}\right)<R_{s},\;\;0\leq ∠T_{k}s_{i}C<\frac{\theta }{2}\\ 0, & \text{other}. \end{array} \end{array}$$

If the interested target was located in one FoV disk of some node and not covered by this node in present, there is the possibility that this target could be covered by this node when proceeding with its self-direction. Taking into account decline of monitoring quality followed by increasing distance between interested target and node, along with the deflected angle between the interested target and current sensing direction of node (*∠T*_*k*_ *s*_*i*_ *C* as shown in Figure 2), the coverage expectation value of node to interested target is defined.

**Definition 4 .**

Expectation value of multimedia sensor node to target *EC*_*T*_*k*__(*s*_*i*_): equation (2) shows coverage expectation value of node *s*_*i*_ to target *T*_*k*_:

(2) ECTk(si) ={λ(1−d(si,Tk)Rs)+β(1−∠TksiCπ),  d(si,Tk)<Rs, 0≤∠TksiC≤πλ(1−d(si,Tk)Rs)+β(1−2π−∠TksiCπ),  d(si,Tk)<Rs, π<∠TksiC<2π0, d(si,Tk)≥Rs,

where  *∠T*_*k*_ *s*_*i*_ *C* means current sensing direction to interested target anticlockwise, with value between [0,2*π*) and parameter 0 ≤ *λ* ≤ 1, 0 ≤ *β* ≤ 1, which defined the adjustable weight of diverted angle and distance between target and node in coverage expectation.

## 4. Self-Directed Target Coverage Optimization

### 4.1. Single-Node Single-Target Self-Direction

At first, single-node single interested target coverage, the simplest circumstance, is discussed. The hypothesis is that interested target was located in FoV disk of node but not covered by node, which needs self-directed adjustment to be covered (Algorithm 1). As long as the target was located in FoV disk, it does not need to regard the distance between target and node in single-node single-target self-direction model. The purpose is the minimal rotated angle of node to realize optimal coverage to interested target which is located in *s*_*i*_ *C*, sensing direction of node.

### 4.2. Multinode Single-Target Self-Directed Coverage

The problem of multinode single-target self-direction is existence of multiple multimedia sensor nodes. The hypothesis is that interested target is not covered by any node while all nodes can cover the interested target through self-direction. According to the coverage expectation value *EC*_*T*_*k*__(*s*_*i*_) of multimedia sensor node to target, the node with maximal coverage expectation value is chosen to proceed with self-direction and cover interested target, which is located in sensing direction of node, in optimal angle. The distance between interested target and node and the angel adjusted during self-direction must be considered (see Algorithm 2).

### 4.3. Single-Node Multitarget Self-Directed Optimal Coverage

The problem of single-node multitarget direction is how to cover maximal interested targets in node self-direction in the circumstance of multiple interested targets randomly located in FoV disk of some node (see Algorithm 3). The central idea of the algorithm is how to select the interested target subset, which is the maximal subset of the difference between any two targets deflection angles less than or equal to FoV vertex angle, as the target of node self-directed coverage optimization when measured by deflection angle of interested target to node current sensing direction.

### 4.4. Multinode Multitarget Self-Directed Coverage Optimization

The problem of multinode multitarget self-directed coverage optimization in WMSN is how to find the minimal node set *S*′ covering a definite interested target set *T* after self-direction. According to coverage corresponding relationship after optimization, the self-direction is proceeded to cover the target node. For expediently showing the self-direction (direction and angle of rotation) of every node, the variance is introduced.

Node *s*_*i*_ rotates to direction *j* in angle of *Q*, 1 as clockwise, 0 as anticlockwise, or 0

The multinode multitarget self-directed coverage optimization can be described as follows:

(3)  Minimize |S′|

(4)  Subject  to t≺S′, ∀t∈T,  S′⊆S

(5) $$\begin{array}{c} \underset{j=0,1}{\sum }\;\underset{0\leq \vartheta \leq \pi }{\sum }\text{ROTAT}{\text{E}}_{ij\vartheta }\leq 1,\;\forall s_{i}\in S^{\prime }, \end{array}$$

where *t*≺*S*′ is *t* falling in ⋃_*s*_*i*_∈*S*′_FoV  After  Rotate_s_i__  , which means that interested target *t* is covered after node set *S*′ self-direction. Objective function (3) is minimal subset of multimedia sensor node which can cover all targets after self-direction. Objective function (4) is reciprocal value of coverage expectation for minimal node subset to all targets. Restrictive condition (5) promises that every interested target can be covered by node set *S*′ after self-direction. Restrictive condition (5) promises that every sensor node *s*_*i*_ ∈ *S*′ can only rotate to one direction at a given angle at any time.

**Figure 3:**
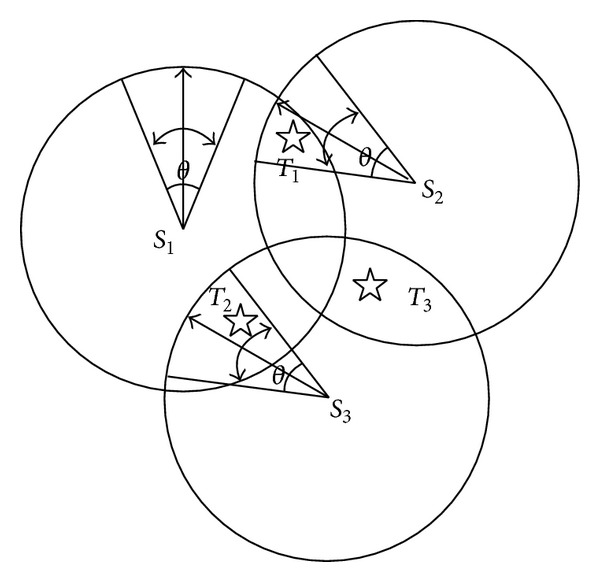
A trinode tri-interested target multimedia sensor network.

**Figure 4:**
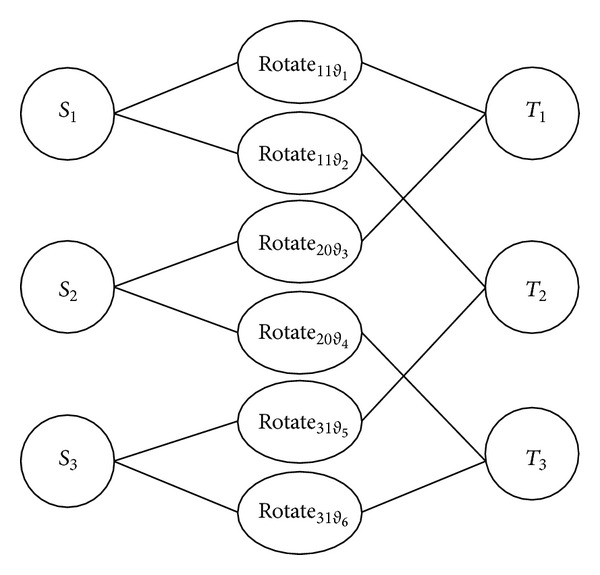
Dilatation figure of Figure 3.

To be described more vividly, a dilatation figure of multinode multitarget self-directed coverage optimization based on simple example in Figure 3 is shown in Figure 4. In literate [11, 12], the potential sensing direction of every node is fixed. But, in this algorithm, every node can define its potential sensing direction according to node covered by its FoV disk, which is named as self-direction. The top of figure includes multimedia sensor node, self-direction action, and interested target. The left side column represents node set *S*. The middle column is self-direction action when every node is covering interested target in its own FoV disk. The right column is interested target set *T*. Every multimedia sensor node *S*_*i*_ is connected with its own self-direction action. Every node can only choose one side because every multimedia sensor node can only do one self-direction action at one time. Every interested target connects with corresponding self-direction action, which means some node can cover this target after self-direction.

Genetic algorithm is used. According to the methods used in [11, 13], the random algorithm in Algorithm 4 is used at first to generate initiate population. It means to generate all possible paths of node set covering all targets through self-direction action based on dilatation figure.

Random algorithm showed in Algorithm 4 may generate empty set. But through executing this algorithm repeatedly, a bigger set of feasible paths in dilatation can be generated, which can be used as initiate group for genetic algorithm. Discriminate algorithm based on genetic algorithm is shown in Algorithm 5.

## 5. Simulation Analysis

**Figure 5:**
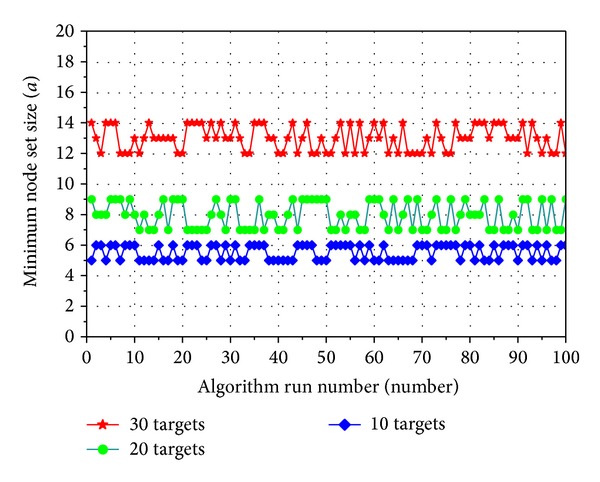
Stability of algorithm.

Simulation analysis is performed to multinode multi-interested target self-direction optimal coverage discriminate algorithm based on genetic algorithm [14]. The MATLAB 7.6.0 and genetic algorithm toolbox function are used. *N* multimedia sensor nodes and *m* interested targets are randomly sown into 400 m × 400 m area. Sensing direction of node is randomly distributed in [0,2*π*] and every interested target should be covered by FoV disk of at least one node. While simulation, the number of sensor nodes is set as 100, sensing angle of sensing node is set as *π*/3, and sensing radius is set as 100 m. Ability of algorithm with 10, 20, and 30 interested targets is investigated individually. Algorithm runs 100 times under every circumstance.  Figure 5 shows the stability of multitarget self-direction coverage optimization by this algorithm under three circumstances. Floating error range of optimal results is less than 3 nodes. The floating range of algorithm running results increased slightly with augmented interested target.

**Figure 6:**
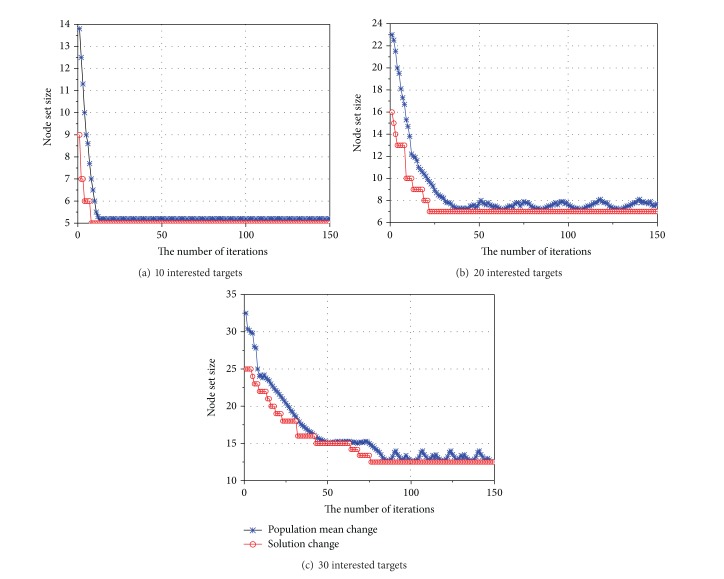
Optimal solution of target function and ability tracking of algorithm.

**Figure 7:**
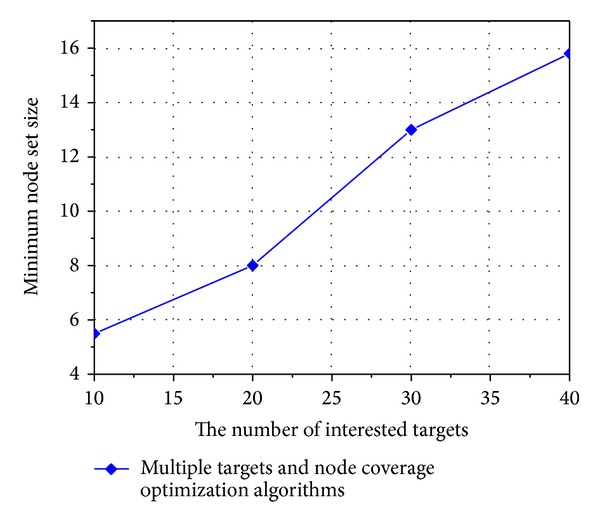
Relationship between number of interested targets and optimal solution of target function.

Figure 6 shows optimal solution of target function and ability tracking of algorithm in different number of interested targets. It can be discerned that our algorithm can generate optimal solution with fewer iteration frequencies, which would increase with augmented interested target.  Figure 7 shows the relationship between number of interested targets and optimal solution of target function. The size of minimal node set which can totally monitor interested targets increases linearly with increasing in number of interested targets. Every data in figure means average value of algorithm ran 100 times.

## 6. Conclusion

In this paper, the factor of rotation angle, rotation direction, and distance between node and interested target when multimedia sensor node self-directed cover interested target in self-directed MSN is discussed. Also we investigate single-node single-target, multinode multitarget, and single-node multitarget self-directed coverage optimization, so node can self-direct in maximal coverage expectation value, which means covering more closer targets with lesser rotation angle. For multinode multitarget self-directed coverage optimization, the hypothesis that all nodes have fixed sensing direction in literature [11] is abandoned. The potential self-directed sensing direction in node is defined by number of interested targets in its FoV disk, which would more fit the feature of randomly deployed WMSN. Then the problem is abstracted into optimization problem with restrictive conditions. Genetic algorithm is used to discriminate. Also simulation analysis is performed. Further study may focus on how to realize multinode multitarget self-directed coverage optimization with induction of coverage expectation based on minimal node covering interested target.

## Figures and Tables

**Algorithm 1:**
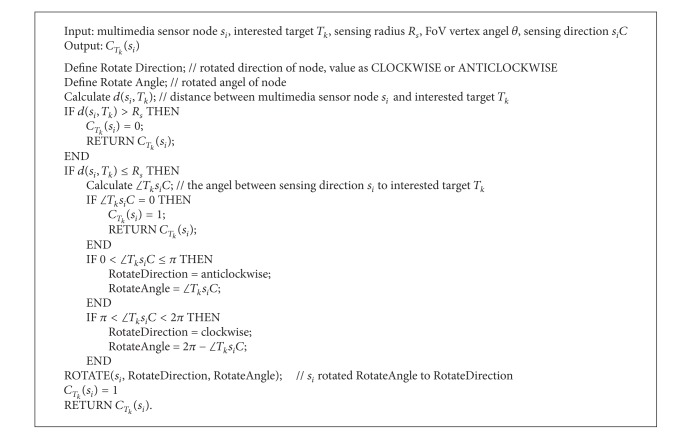
Single-node single-interested target self-direction coverage algorithm.

**Algorithm 2:**
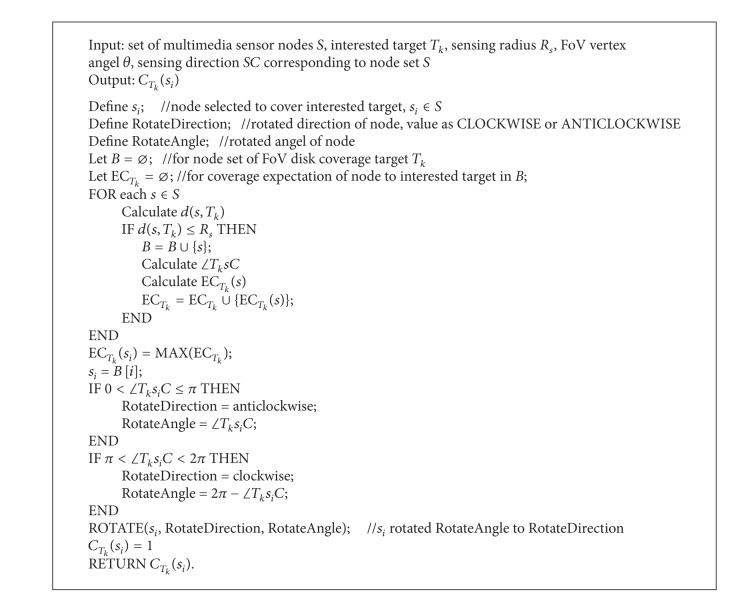
The algorithm of multinode single-target self-directed coverage.

**Algorithm 3:**
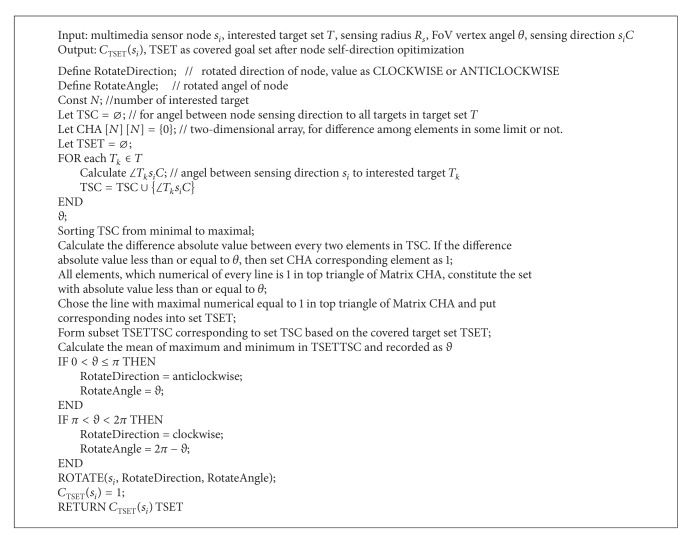
Algorithm of single-node multi-interested target self-direction optimal coverage.

**Algorithm 4:**
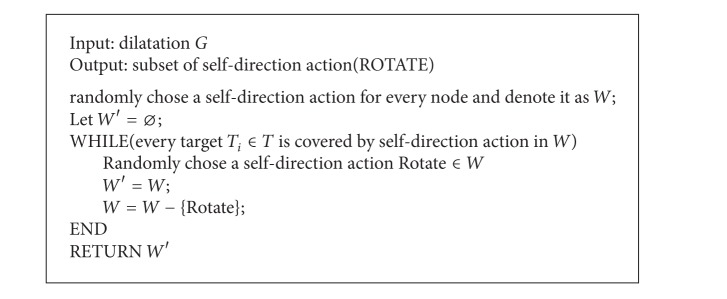
Random algorithm to generate all possible paths in dilatation *G*.

**Algorithm 5:**
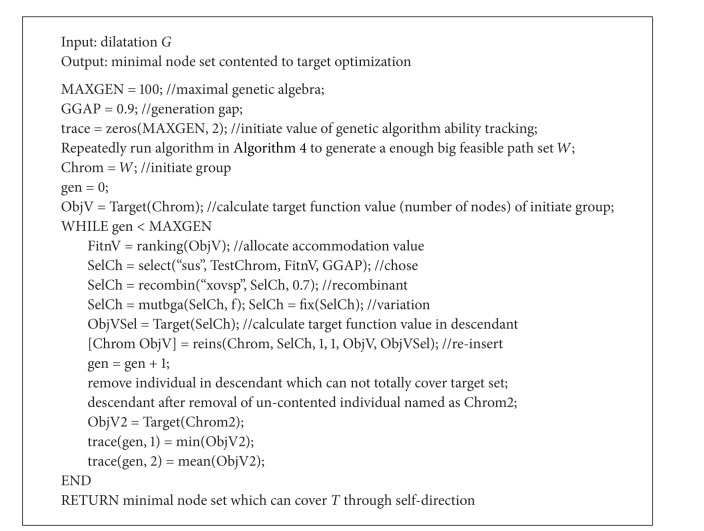
Multinode multi-interested target self-direction optimal coverage discriminate algorithm based on genetic algorithm.
